# Trifecta of Cysts: Unraveling the Diagnosis in a Patient With Renal, Hepatic, and Pancreatic Cysts

**DOI:** 10.7759/cureus.88665

**Published:** 2025-07-24

**Authors:** Arielle N Washington, Kyle W Plunk, Jesus E Guarecuco Castillo, Nir Hus

**Affiliations:** 1 General Surgery, St. George's University School of Medicine, St. George's, GRD; 2 Surgery, Larkin Community Hospital, South Miami, USA; 3 Surgery, Florida Atlantic University, Boca Raton, USA; 4 Surgery, Delray Medical Center, Delray Beach, USA

**Keywords:** benign pancreatic cystic lesions, multiorgan involvement, multiple cysts, multiple hepatic cysts, renal cysts

## Abstract

Multiorgan cysts, affecting multiple organs such as the liver, kidneys, and pancreas, pose a diagnostic and therapeutic challenge. The etiology is diverse, including congenital conditions, like polycystic kidney disease (PKD) and von Hippel-Lindau (VHL) syndrome, as well as secondary causes, like infection or neoplasms. The clinical presentation ranges from incidental findings to life-threatening complications. This case report discusses a 65-year-old female patient who presented with abdominal pain and was incidentally found to have hepatic, renal, and pancreatic cysts. Diagnostic workup, including computed tomography (CT), magnetic resonance imaging (MRI), and laboratory tests, ruled out primary genetic conditions, and management involved conservative monitoring and surgical interventions for bile leakage. The case emphasizes the complexity of diagnosing and managing multiorgan cysts and the importance of multidisciplinary care.

The report highlights the need for further research to enhance diagnostic accuracy and improve therapeutic strategies in managing multiorgan cysts.

## Introduction

Multiorgan cysts, characterized by cystic formations in multiple organs, represent a complex and often challenging clinical scenario. These cysts can be congenital or acquired and may affect various organs, including the liver, kidneys, pancreas, spleen, and lungs. The causes of multiorgan cysts can vary widely, including genetic disorders, like polycystic kidney disease (PKD) and von Hippel-Lindau (VHL) syndrome, as well as secondary factors, such as infections, tumors, or inflammation. 

The clinical presentation of multiorgan cysts varies widely, depending on the number, size, and location of the cysts and the degree of organ involvement. Symptoms can range from asymptomatic findings discovered incidentally on imaging studies to severe, life-threatening complications such as infection, bleeding, or organ failure. Due to the potential for significant morbidity, early recognition and appropriate management of multiorgan cysts are crucial. 

Diagnosis typically involves a combination of imaging modalities, including ultrasound, computed tomography (CT), and magnetic resonance imaging (MRI), which provides detailed information about the cystic structures and their impact on surrounding tissues. Genetic testing and family history can also play a pivotal role in identifying hereditary syndromes associated with cyst formation.

Management strategies for multiorgan cysts are tailored to the individual patient and the cysts' specific characteristics. Treatment options may include conservative monitoring, pharmacotherapy aimed at slowing cyst progression, minimally invasive procedures such as aspiration or sclerotherapy, and surgical interventions for more complex or symptomatic cases [1]. Multidisciplinary care involving nephrologists, hepatologists, gastroenterologists, and surgeons is often required to optimize outcomes.

This case report discusses a patient presenting with multiorgan cysts, detailing the diagnostic workup, clinical course, and therapeutic interventions undertaken. The report aims to highlight the complexities of managing multiorgan cysts and contribute to the existing body of knowledge on this multifaceted condition.

## Case presentation

**Figure 1:**
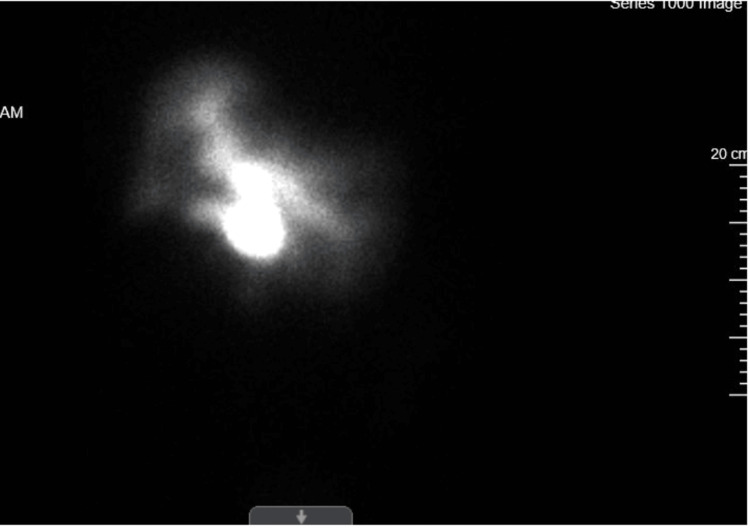
HIDA scan completed on October 14, 2023, suggestive of bile leak HIDA: hepatobiliary iminodiacetic

A 65-year-old female patient with a past medical history of robotic cholecystectomy presented to the emergency room with complaints of abdominal pain, with an incidental CT scan revealing multiorgan cysts in the liver, kidneys, and pancreas. Initially, the patient presented to the emergency room complaining of abdominal pain, and a hepatobiliary iminodiacetic (HIDA) scan was ordered due to suspicion of a bile leak after a recent cholecystectomy. The HIDA scan revealed extravasation of the isotope in the gallbladder fossa, suggesting a bile leak (Figure 1). Based on imaging findings, the patient received an endoscopic retrograde cholangiopancreatography (ERCP) and stent placement to correct the bile leak. 

**Figure 2:**
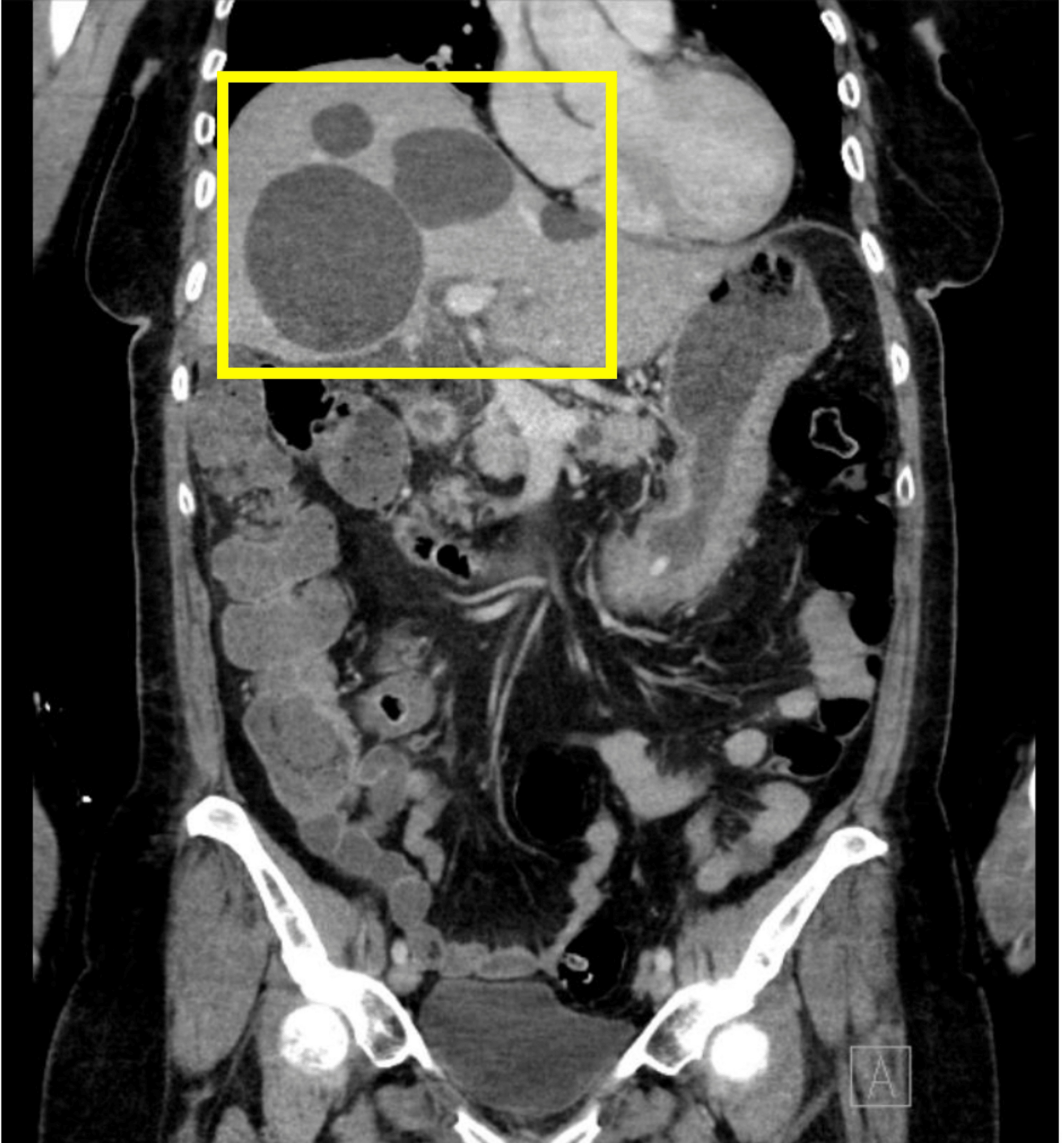
Multiple hepatic cysts labeled with a yellow square on CT scan CT: computed tomography

However, three days after discharge, the patient presented to the emergency room again for diffuse abdominal pain, decreased appetite, and nausea without fever, vomiting, or dysuria, with abdominal tenderness on palpation, specifically in the periumbilical region. In order to rule out other pathologies, patient labs were ordered, revealing the following: WBC 14.8×1000 WBCs/μL, neutrophils 89.1 WBCs/μL, hemoglobin (Hgb) 12.2 g/dL, hematocrit (Hct) 37.1, and platelets 429,000. Due to the patient's presentation, an abdominal CT scan was ordered, which initially revealed multiple hypodense lesions throughout the liver, the largest in the right hepatic lobe measuring approximately 8 cm, probably cysts (Figure 2).

**Figure 3:**
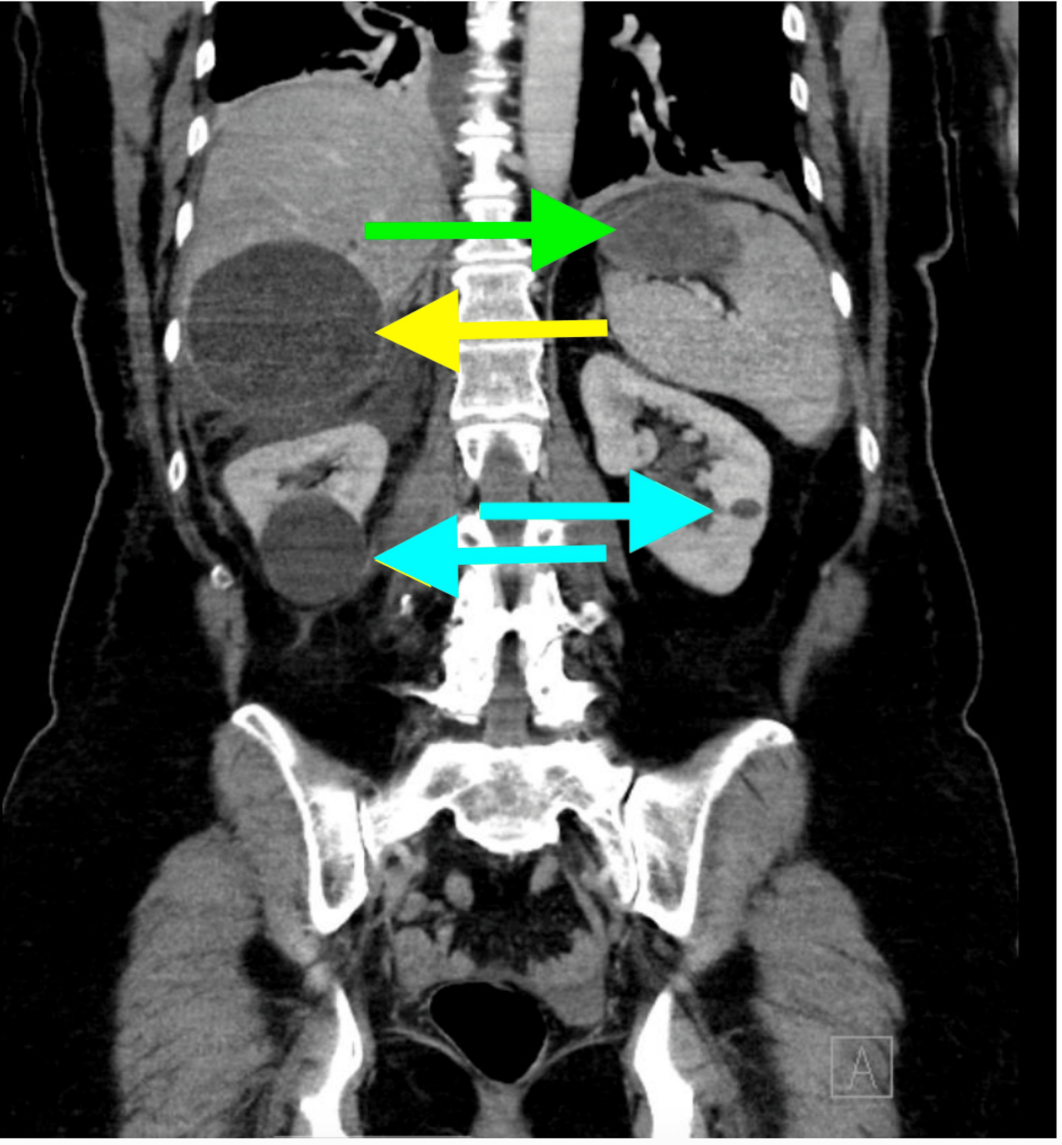
Splenic, hepatic, and renal cysts indicated by green, yellow, and blue arrows, respectively, on CT scan CT: computed tomography

The CT scan study reports the following additional findings: (a) multiple hypodense lesions in the body and tail of the pancreas, the largest measuring 1.6 cm (Figures 2-3); (b) average renal size and position; (c) no hydronephrosis; (d) symmetric nephrograms; (e) right renal cyst measuring 4.2 cm (Figure 3); (f) multiple left renal cysts, the largest measuring 1 cm (Figure 3); (g) a final impression of multiple hepatic and bilateral renal cysts; and (h) numerous cystic lesions in the pancreas as well. It cannot exclude cystic neoplasm. There was a radiology recommendation for further evaluation with MRI for optimal characterization. 

**Figure 4:**
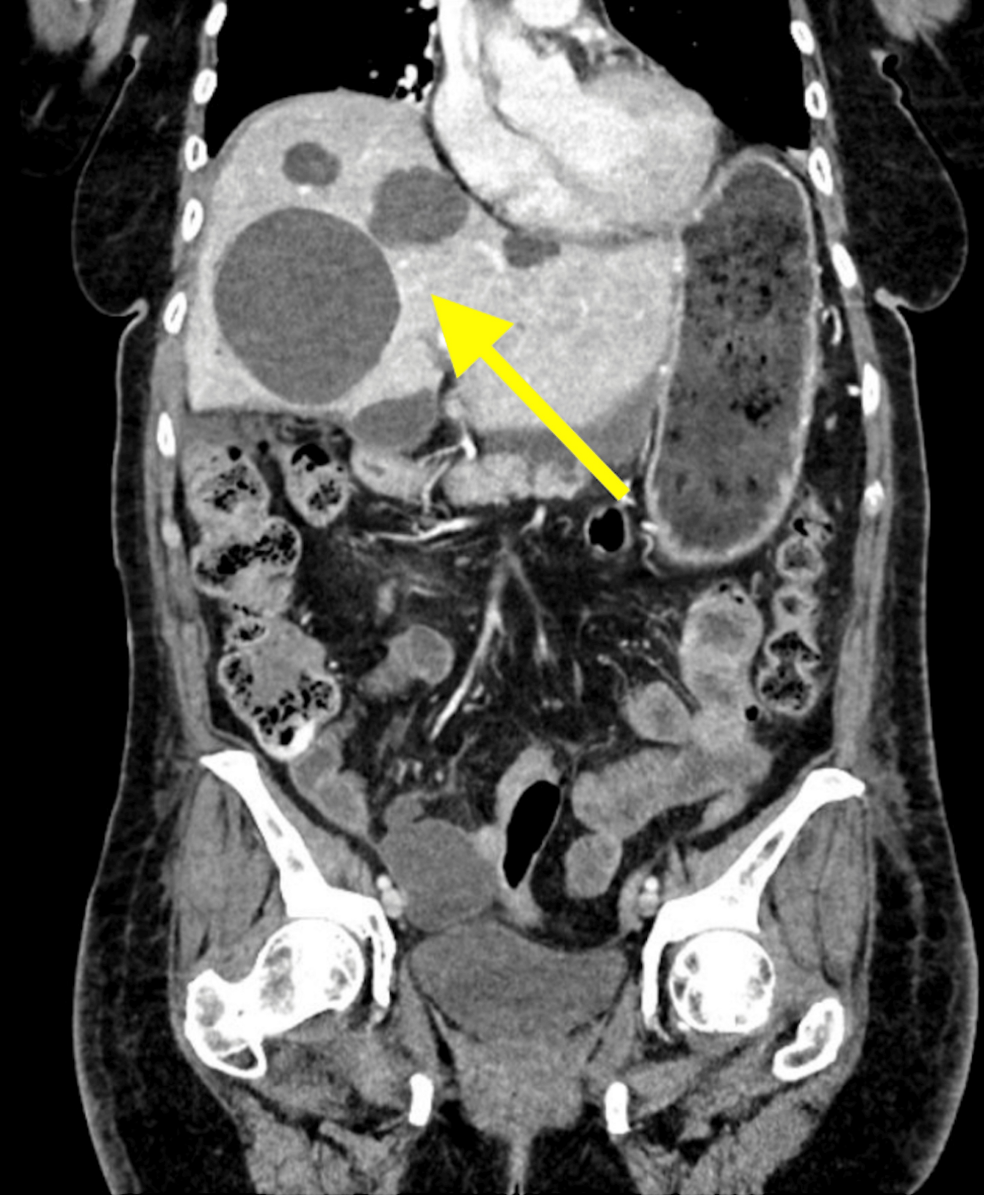
Multiple hepatic cysts indicated by a yellow arrow on CT scan CT: computed tomography

**Figure 5:**
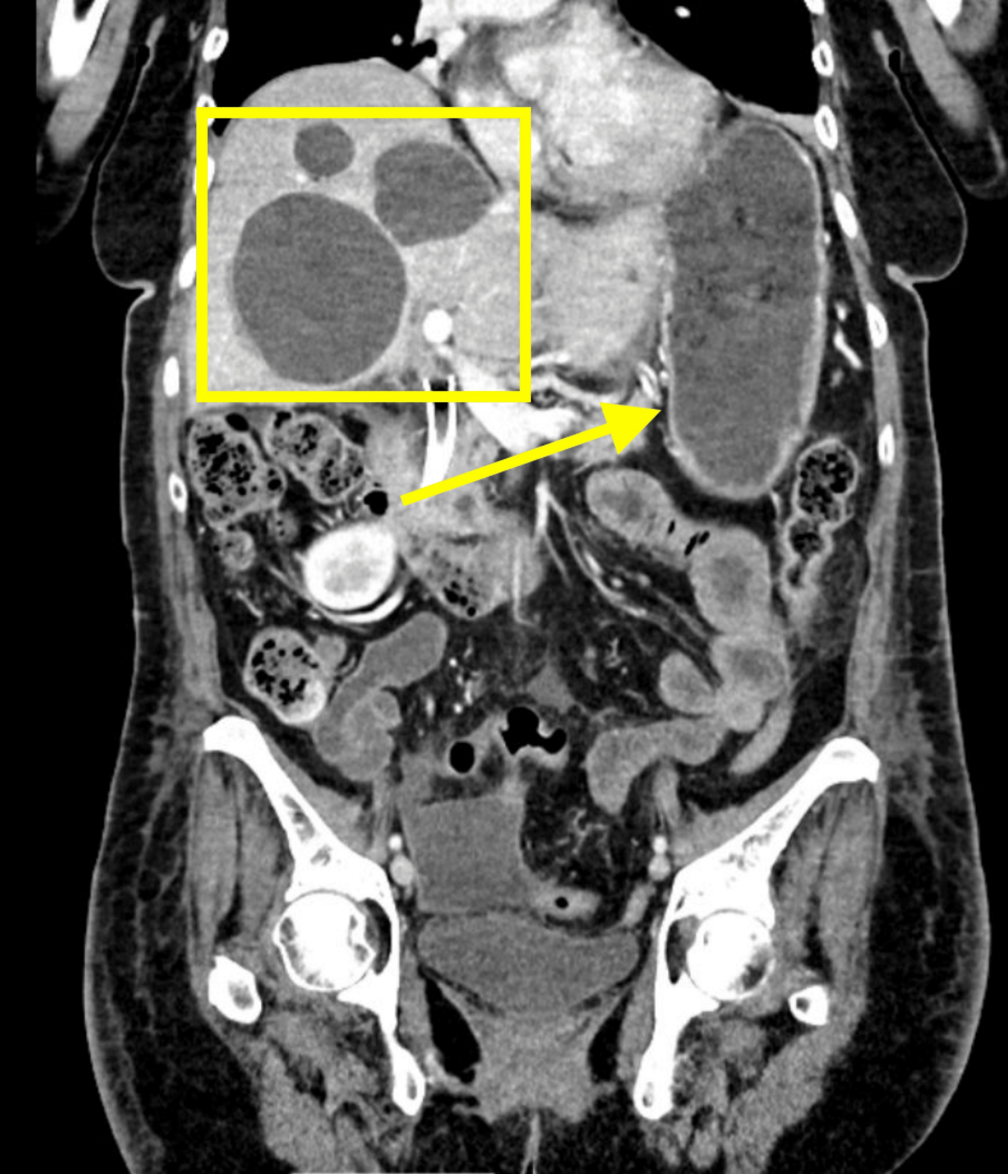
Multiple hepatic cysts indicated by a yellow square and splenic cyst indicated by a yellow arrow on CT scan CT: computed tomography

Abdominal CT scan follow-up 16 days after the initial CT demonstrated improvement of pelvic ascites. Multiple hepatic cysts were re-demonstrated, the largest of which measured 7 cm (Figure 4). Additionally, the gallbladder was absent with the presence of a stable common duct stent in an adequate position. Several subcentimeter pancreatic cystic lesions were again noted (Figure 5). The kidneys excrete the IV contrast symmetrically without evidence of hydronephrosis caused by a 3 cm right renal cyst (Figure 4).

A magnetic resonance cholangiopancreatography (MRCP) was ordered due to generalized abdominal pain and revealed multiple cysts within the liver without evidence of hepatic steatosis. The findings of the MRCP are as follows: presence of a cortical cyst arising from the lower pole of the right kidney, no visualized gallbladder, no intrahepatic ductal dilation, and the common bile duct measuring 7.5 mm, with signal dropout in the distal common bile duct in the patient's intrahepatic stent region. Imaging also noted two contiguous cysts in the body of the pancreas measuring 1.2 cm and 0.9 cm in the greatest dimension, which could represent a septated cyst. Radiology recommended follow-up to ensure stability. Tiny cysts in the tail of the pancreas could not be evaluated in this study. There was no definitive evidence for choledocholithiasis.

Diagnostic modalities

Diagnosing hepatic cysts relies on clinical, laboratory, and imaging findings. Blood cultures, liver function tests, and inflammatory markers contribute to the diagnostic workup. Imaging modalities such as ultrasound, CT, and MRI are needed to confirm the diagnosis and assess cyst size and location.

## Discussion

Multiorgan cysts may present with a spectrum of symptoms, ranging from nonspecific constitutional symptoms, such as fever and malaise, to more localized signs, including right upper quadrant pain, hepatomegaly, and jaundice. The clinical presentation can vary based on the underlying etiology. In this case, the multiorgan cysts were found incidentally on a CT scan, with the only clinical clue presenting as diffuse abdominal pain. The path to this unique diagnosis began with conducting laboratory and imaging studies to rule out other potential pathologies such as hepatic cysts, PKD, and VHL syndrome.

Hepatic cysts can be classified into pyogenic, amebic, and fungal based on their causative agents. Pyogenic cysts often result from bacterial dissemination, with the most common pathogens being *Escherichia coli* and *Klebsiella pneumoniae*. Amebic cysts are predominantly caused by the protozoan parasite *Entamoeba histolytica*, while fungal cysts may occur in immunocompromised individuals. In this case, hepatic cysts were ruled out due to a lack of laboratory evidence of infection and negative imaging [2]. 

PKD is a chronic, multisystem disease commonly involving the kidneys, leading to bilateral kidney cyst formation. The inheritance pattern further subdivides into autosomal dominant and recessive types, with the dominant type being more prevalent in adults. Autosomal dominant polycystic kidney disease (ADPKD) was a potential diagnosis in this case due to its feature of multiorgan cyst involvement. ADPKD can present with extrarenal cysts involving the liver, pancreas, and spleen, as seen in our patient [3]. This condition is often diagnosed based on laboratory findings indicating progressive kidney damage and hypertension, ultrasound imaging revealing renal cysts, and a positive family history due to its strong genetic component [3]. However, this patient was normotensive with normal glomerular filtration rate and creatinine levels as well as a negative family history, thus ruling out the potential diagnosis of ADPKD. 

Another considered diagnosis was VHL syndrome, a multisystem inherited disorder characterized by cysts, hemangioblastomas, renal cell carcinoma, and pheochromocytoma. VHL syndrome presents with symptoms of cyst enlargement, which can include vague abdominal pain, fundoscopy of abnormal retinal findings, and laboratory findings of elevated urine catecholamines with episodic headaches, panic attacks, and elevated blood pressure consistent with a diagnosis of pheochromocytoma [4]. In this case, our patient did not meet the criteria for any conditions necessary to diagnose VHL syndrome.

Splenic cysts are fluid-filled sacs located within the spleen, frequently discovered incidentally during imaging studies conducted for unrelated conditions. These cysts can be categorized as primary or secondary; primary cysts are typically congenital, including epidermoid and dermoid types, while secondary cysts may result from traumatic or infectious processes, such as hydatid disease. Most splenic cysts are asymptomatic and require no treatment; however, larger cysts or those that cause symptoms may require management. Non-surgical approaches often involve observation and periodic imaging to monitor the cyst's size and symptomatology. Surgical management may include laparoscopic or open splenectomy, especially for cysts that are symptomatic, large, or suspected to be malignant. Understanding the etiology and potential complications associated with splenic cysts is essential for accurate diagnosis and effective management [5].

Multiorgan cysts can lead to various complications, including sepsis, cyst rupture, and systemic dissemination of infection. Close monitoring and timely intervention are crucial to mitigate these complications and improve patient outcomes. The prognosis of multiorgan cysts depends on the underlying etiology, timely diagnosis, and appropriate management. With prompt and effective treatment, many patients can achieve a full recovery. However, delayed diagnosis or inadequate therapy may lead to significant morbidity and mortality.

## Conclusions

Multiorgan cysts require a multidisciplinary approach involving clinicians, radiologists, and surgeons. Early recognition, accurate diagnosis, and appropriate management are pivotal for optimal patient outcomes. Further research is needed to refine diagnostic and therapeutic strategies and enhance our understanding of the evolving epidemiology of multiorgan cysts.
